# Permanent Hypoparathyroidism After Total Thyroidectomy in Children: Results from a National Registry

**DOI:** 10.1007/s00268-018-4552-7

**Published:** 2018-02-22

**Authors:** Erik Nordenström, Anders Bergenfelz, Martin Almquist

**Affiliations:** Department of Clinical Science, Lund University, 221 85 Lund, Sweden

## Abstract

**Introduction:**

Hypoparathyroidism is the most common complication following thyroidectomy. There are few population-based reports on the rate of hypoparathyroidism in children. The incidence of medical treatment of permanent hypoparathyroidism in children is reported using a national registry.

**Methods:**

The study population included patients below 18 years of age undergoing total thyroidectomy reported to the Scandinavian Quality Registry for Thyroid, Parathyroid and Adrenal Surgery 2004–2014. Patients with previous thyroid or parathyroid surgery or treatment with vitamin D before surgery were excluded from analysis. Permanent postoperative hypoparathyroidism was defined as treatment with vitamin D for more than 6 months after thyroidectomy. Risk factors for permanent hypoparathyroidism were calculated with uni- and multivariable logistic regression. Using data from the Swedish Inpatient Registry, rates of readmissions and annual number of days in hospital after total thyroidectomy were compared between patients with and without permanent hypoparathyroidism.

**Results:**

Some 274 children (215 girls and 59 boys) underwent total thyroidectomy. The median age was 14 (range 0–17) years. Indications for surgery were Graves’ disease (214, 78.1%), other benign disease (27, 9.9%) and thyroid cancer (33, 12%). Median follow-up was 4.8 years. Twenty (7.3%) children developed permanent hypoparathyroidism. No statistically significant risk factors for permanent hypoparathyroidism were identified. Rates of readmission and annual number of days in hospital after discharge were similar in patients with and without permanent hypoparathyroidism.

**Conclusions:**

The rate of permanent hypoparathyroidism following total thyroidectomy in children was high and is a cause of concern.

## Introduction

Hypocalcemia is the most common complication after total thyroidectomy [1–3]. Reports have shown that pediatric patients undergoing thyroid surgery have higher incidence of complications compared to adults even in high-volume centers [4–7]. Few population-based register studies have described the rate of postoperative permanent hypocalcemia and the consequences of this condition in children. One population-based study on thyroid cancer has indicated very high frequency of permanent hypoparathyroidism following thyroid surgery in children [8].

It was hypothesized that the rate of permanent hypocalcemia following total thyroidectomy in children would be more accurately analyzed by combining data from national registries. Additionally, this study evaluated the rates of readmission and annual number of days in hospital after discharge. The outcome after thyroidectomy in Swedish children registered in the Scandinavian Quality Register for Thyroid, Parathyroid and Adrenal Surgery (SQRTPA), the Swedish National Registry for Prescribed Drugs, and the Swedish National Inpatient Register were studied.

## Methods

### Data sources

#### SQRTPA

The Scandinavian Quality Registry for Thyroid, Parathyroid and Adrenal Surgery (SQRTPA, www.sqrtpa.se) started in 2004 and is recognized by the Swedish National Board for Health and Social Welfare as the national quality registry for endocrine surgical procedures in Sweden. Data are entered online prospectively by the surgeon or a specialized nurse. Currently, 36 Swedish units report to the registry, covering almost 100% of all thyroid procedures in Sweden [2, 9]. Coverage is assessed by calculating the proportion of patients registered in SQRTPA in relation to those registered in the Swedish National Patient register. Data quality for the registered patients is evaluated by external audit and by comparing registered data to hospital medical records. The audit has demonstrated good data quality, with an error rate of <5% [9].

### Swedish National Registry for Prescribed Drugs

The Swedish Prescribed Drug Register (SPDR) was launched in 2005 and contains information about age, sex and unique identification of the patient, Anatomical Therapeutic Chemical (ATC) Classification Code, drug dose, package size, date for prescription, day when the drug was bought at the pharmacy, as well as the prescriber’s profession and practice. The data are entered in an automatical fashion by direct transfer from the computer record. The prescribed drugs included in the register, accounted for 84% of the total utilization [10].

### The Swedish National Patient register

As of 1987, the Swedish National patient register (NPR) includes all in-hospital patient care in Sweden. The number of surgical procedures and diagnoses at all hospitals in Sweden can be retrieved from this register. Data are entered by direct transfer from computer record system in each county. Underreporting for inpatient data has been estimated to less than one percent [11].

### Study cohort

Patients below the age of 18 operated with total thyroidectomy January 1, 2004 to December 31, 2014 were identified in the SQRTPA.

The primary endpoint was the incidence of permanent postoperative hypoparathyroidism defined as medication with active vitamin D for more than 6 months after thyroidectomy in the SPDR.

Exclusion criteria were previous thyroid and parathyroid surgery and ongoing treatment with vitamin D at the time of surgery. Permanent hypoparathyroidism was defined as prescription and expedition of active vitamin D (dihydrotachysterol, ATC A11CC02, alfacalcidol, ATC A11CC03 or calcitriol, ATC A11CC04) in the first 180 days after surgery and any time after 180 days after surgery. Data on age, sex, indication for thyroidectomy, and lymph node dissection, number of identified parathyroid glands, parathyroid auto transplantation, operation time, thyroid specimen weight, and serum calcium at discharge and at 6 weeks postoperatively were retrieved. Surgical volume and the number of in-patient events including the diagnosis were extracted from the NPR.

Hospital volume of thyroid surgery was defined as low when less than 50 operations were done per year, median when 50–100, and high when more than 100 thyroid operations were performed annually.

### Statistical analysis

Descriptive statistics were performed, and results calculated as medians with interquartile ranges (IQR) and numbers with column percentages. Differences between groups were evaluated with Mann–Whitney and Chi-square or Fishers exact test, where appropriate.

The risk of permanent hypoparathyroidism was investigated with uni- and multivariable logistic regression, yielding odds ratios (OR) with 95 percent confidence intervals (CI). The multivariable model included factors deemed to potentially affect risk of hypoparathyroidism: age at surgery (continuous), sex, hospital volume in categories, type of thyroid disease, number of identified parathyroid glands, parathyroid auto transplantation (yes or no), operation time (in categories), thyroid specimen weight (in categories) and whether lymph node dissection was performed or not. All statistical analyses were made using STATA version 12 (StataCorp LP, College station, USA).

### Ethical considerations

The ethical committee at Lund University approved the study (2011/740, 2015/543 and 2016/83).

## Results

Some 274 children (215 girls and 59 boys) undergoing total thyroidectomy were included in the study. The median age was 14 (range 0–17) years. Indications for surgery were Graves’ disease (214, 78.1%), other benign disease (27, 9.9%) and thyroid cancer (33, 12%). Median follow-up was 4.8 years. Median follow-up was 4.8 years. Twenty patients (7.3%) developed permanent hypoparathyroidism according to study definition. Descriptive statistics for patients with or without permanent hypoparathyroidism are shown in Table 1. There were no statistically differences regarding age, type of thyroid disease, autotransplantation of parathyroid glands, lymph node surgery, thyroid specimen weight, length of hospital stay or hospital volume of thyroid surgery between patients with or without permanent hypoparathyroidism. There was, however, a difference in operation time. Serum calcium was lower at discharge and 6 weeks after surgery in patients with permanent hypoparathyroidism.

**Table 1:** Total thyroidectomy in children: descriptive statistics

	No permanent hypoparathyroidism (*n* = 254, 93%)	Permanent hypoparathyroidism (*n* = 20, 7%)	*p*
Age	15 (13–17)	13 (10–16)	0.09
Sex			
Male	199 (78)	16 (80)	
Female	55 (22)	4 (20)	0.86
Type of thyroid disease			
Benign^a^	25 (10)	2 (10)	
Thyrotoxicosis	199 (78)	15 (75)	
Malignant	30 (12)	3 (15)	0.91
Preoperative calcium level (mmol/L) (reference 2.20–250 mmol/L)	2.37 (2.29–2.44)	2.37 (2.23–2.44)	0.97
Number of identified parathyroid glands			
0	1 (0)	0 (0)	
1	9 (4)	1 (5)	
2	53 (21)	6 (30)	
3	90 (35)	6 (30)	
4 or more	101 (40)	7 (35)	0.88
Parathyroid re-implantation			
No	160 (63)	12 (60)	
Yes	94 (37)	8 (40)	0.79
Lymph node surgery			
No	218 (86)	18 (90)	
Yes	36 (14)	2 (10)	0.60
Operation time (min)			
<120	67 (26)	6 (30)	
120–180	103 (41)	5 (25)	
>180	26 (10)	6 (30)	
Not reported	58 (23)	3 (15)	0.047
Thyroid specimen weight, grams			
<25	51 (20)	6 (30)	
25–50	83 (33)	2 (10)	
>50	47 (18)	5 (25)	
Not reported	73 (29)	7 (35)	0.20
Serum calcium at discharge (mmol/L)^b^	2.11 (2.00–2.23)	1.78 (1.63–1.98)	<0.001
Serum calcium at 6 weeks (mmol/L)	2.34 (2.28–2.40)	2.14 (2.12–2.21)	<0.001
Hospital stay (days)	1 (0–2)	2 (0–4)	0.40
Hospital volume			
Low (<50)	33 (13)	4 (20)	
Median (50–100)	40 (16)	3 (15)	
High (>100)	181 (72)	13 (65)	0.68

*N* (column percent) and Chi-square or Fishers exact where appropriate; median (IQR) and Mann–Whitney or Wilcoxon rank sum test

^a^Benign neoplasm and multinodular colloid goiter

^b^Reference range 2.20–2.50 mmol/L

Uni- and multivariable regression analyses of potential risk factors for permanent hypoparathyroidism are shown in Table 2. No statistically significant risk factors for permanent hypoparathyroidism were identified. The total number of operations in children per hospital in relation to the annual hospital volume of thyroid surgery during the study period is shown in Table 3. The majority of operations, 194 (70.8%), were performed in high-volume hospitals.

**Table 2:** Risk of permanent hypoparathyroidism after total thyroidectomy

	Univariate regression	Multivariate regression
	OR (95% CI)	OR^b^ (95% CI)
Age	0.90 (0.81–1.02)	0.89 (0.78–1.01)
Gender		
Female	1.00	1.00
Male	0.90 (0.29–2.82)	0.73 (0.19–2.75)
Type of thyroid disease		
Thyrotoxicosis	0.94 (0.20–4.36)	1.38 (0.22–8.87)
Benign^a^	1.00	1.00
Malignant	1.25 (0.19–8.08)	19.6 (0.6–6.28)
Lymph node surgery		
No	1.00	1.00
Yes	0.67 (0.15–3.02)	0.25 (0.01–7.07)
Number of identified parathyroid glands		
2 or less	1.00	1.00
3 or more	0.61 (0.23–1.60)	0.69 (0.24–2.05)
Parathyroid re-implantation		
No	1.00	
Yes	1.13 (0.45–2.88)	1.34 (0.45–4.02)
Operation time (min)		
<120	1.00	1.00
120–180	0.54 (0.16–1.85)	0.52 (0.14–1.93)
>180	2.58 (0.76–8.72)	4.43 (0.94–20.8)
Not reported	0.58 (0.14–2.41)	0.49 (0.10–2.29)
Thyroid specimen weight (g)		
<25	1.00	1.00
25–50	0.20 (0.04–1.05)	0.22 (0.04–1.23)
>50	0.90 (0.26–3.16)	0.59 (0.13–2.59)
Not reported	0.82 (0.26–2.57)	0.75 (0.21–2.65)
Hospital volume		
Low	1.69 (0.52–5.49)	2.11 (0.62–7.17)
Medium	1.04 (0.28–3.84)	1.32 (0.34–5.18)
High	1.00	1.00

^a^Benign neoplasm and multinodular colloid goiter

^b^Adjusted for all factors in table

**Table 3:** Total number of thyroid operations in children and annual hospital volume for thyroid surgery as categories during the study period

Hospital	Hospital volume			Total
	High (>100/year)	Medium (50–100/year)	Low (<50/year)	
1	41	0	0	41
2	0	2	0	2
3	0	9	0	9
4	28	0	0	28
5	0	0	2	2
6	0	3	0	3
7	0	0	7	7
8	76	0	0	76
9	0	0	3	3
10	49	0	0	49
11	0	2	0	2
12	0	2	0	2
13	0	0	18	18
14	0	7	0	7
15	0	0	8	8
16	0	3	0	3
17	0	0	3	3
18	0	5	0	5
19	0	1	0	1
20	0	2	0	2
21	0	0	1	1
22	0	0	2	2
Total	194	43	37	274

Rates of readmission and annual number of days in hospital after discharge were similar in patients with and without permanent hypoparathyroidism (Table 4).

**Table 4:** Follow-up after total thyroidectomy in children

	No permanent (*n* = 254)	Permanent (*n* = 20)	*p*
Follow-up (years)	4.8 (3.0–6.9)	3.5 (1.3–6.6)	0.63
Readmissions^a^	0 (0–0.1)	0 (0–0.2)	0.84
Days in hospital^a^	0 (0–0.1)	0 (0–0.2)	0.93
Prescriptions^a^	17 (10–27)	22 (15–46)	0.03

^a^Annual number of readmissions, days in hospital and prescriptions per year after discharge from admission for thyroidectomy (i.e., total number/time followed)

## Discussion

When indicated, total thyroidectomy is accepted as the standard surgical procedure for thyroid disease in adults and children [12, 13]. There are few population-based register studies evaluating permanent hypoparathyroidism following total thyroidectomy in children [8]. Some previous studies, mainly single-center studies from specialized institutions, have indicated that thyroid surgery in children is associated with a low complication rate, i.e., permanent hypoparathyroidism of 1.5% [14]. In the present study, the overall frequency of permanent hypoparathyroidism following total thyroidectomy in children was 7.3%. This is a high figure and is a cause of concern. The study was based on combined data from the national surgical quality register (SQRTPA) and the Swedish National Registry for Prescribed Drugs, and therefore, the results should be robust.

A previous population-based study from the Netherlands showed that 25% of pediatric patients undergoing thyroid surgery for differentiated thyroid cancer suffered from permanent hypoparathyroidism [8]. However, some 40% of these patients underwent nodal dissection.

No specific risk factors for permanent hypoparathyroidism were identified in the multivariable logistic regression analysis. Data from numerous studies have indicated that high operation volume is associated with lower complication rate after thyroid surgery [15–18]. However, data on the individual surgeons’ volume could not be analyzed in the present study, since this is not registered in the SQRTPA. Hospital volume of thyroid surgery was not a risk factor for permanent hypoparathyroidism.

One explanation of the high frequency of permanent hypoparathyroidism could be that some patients, in contrary to single centers series, are operated in low volume hospitals. However, the majority of the operations reported in the present investigation were done in high-volume centers which should provide the individual surgeon with an adequate operation volume per year.

The reason for the high risk of permanent hypoparathyroidism in children is unknown. Risk factors for postoperative hypoparathyroidism were recently reviewed [19]. None of the previously identified risk factors for permanent hypoparathyroidism; Graves’ disease, fewer than two parathyroid gland identified during surgery and heavier thyroid specimens were risk factors in the present study.

It is also suggested that children are more difficult to operate anatomically and that surgeon’s volume is the most important factor for outcome. For reasons discussed previously, this was not analyzed in the present study.

Furthermore, measures to prevent permanent hypoparathyroidism were analyzed in another systematic review [20]. However, the result was disappointing, and no specific measure was associated with a decreased risk, although most studies were of low quality. In this study, lymph node (LN) surgery was not associated with an increased risk of permanent hypoparathyroidism. On the contrary, even if not significant OR was less than one, i.e., it seems that it was a trend that LN surgery could protect the patients from hypoparathyroidism. The reason for this is unclear but could be that these procedures were done by the most experienced surgeons and or that when doing LN surgery the surgeon was extra careful to preserve the parathyroid glands. If the frequency of permanent hypoparathyroidism in children is high even in the hands of experienced high-volume surgeons new efforts must be taken to try to change this situation. Maybe new methods such as infrared light during thyroid surgery could be one tool [21]. The frequency of parathyroid autotransplantation was 37–40% in this study. If the frequency of autotransplantation was high (too high?) is hard to comment since we have not found any similar studies describing the frequency of parathyroid autotransplantation in children. Further autotransplantation of the parathyroid glands did not protect patients from permanent hypoparathyroidism in this study, and this is supported from a recent study from Spain [22] that showed that autotransplantation did not influence the rate of permanent hypoparathyroidism.

The long-term consequences of permanent hypoparathyroidism, for instance skeletal development, growth mental health and impact quality of life, results in school among others, were not addressed by the present investigation. In the present investigation, the number of readmissions after surgery and the annual number of days in hospital after discharge was used as a proxy for medium term risk for increased comorbidities. No differences in readmissions or annual days in hospital after the thyroid operation were detected in the two groups of patients with a median follow-up time of almost 5 years. However, future morbidity in children treated due to permanent hypoparathyroidism is a cause for great concern.

A number of limitations of the current study are acknowledged. The number of operations was comparatively small, and although the data quality should be high using national registries, this will certainly influence the possibility to detect important risk factors for permanent hypoparathyroidism. The lack of data for the individual surgeon’s volume is another important drawback of the study that could not be mitigated.

The rate of permanent hypoparathyroidism following total thyroidectomy in children was high in this population-based register study and is a cause of concern.

## References

[1] Rosato L, Avenia N, Bernante P, De Palma M, Gulino G, Nasi PG, Pelizzo MR, Pezzullo L (2004). Complications of thyroid surgery: analysis of a multicentric study on 14,934 patients operated on in Italy over 5 years. World J Surg.

[2] Bergenfelz A, Jansson S, Kristoffersson A, Mårtensson H, Reihnér E, Wallin G, Lausen I (2008). Complications to thyroid surgery: results as reported in a database from a multicenter audit comprising 3660 patients. Langenbecks Arch Surg..

[3] Hallgrimsson P, Nordenstrom E, Bergenfelz A, Almquist M (2012). Hypocalcaemia after total thyroidectomy for Graves’ disease and for benign atoxic multinodular goitre. Langenbecks Arch Surg.

[4] Sinha CK, Decoppi P, Pierro A, Brain C, Hindmarsh P, Butler G, Dattani M, Spoudeas H, Kurzawinski TR (2015). Thyroid surgery in children: clinical outcomes. Eur J Pediatr Surg.

[5] Scholz S, Smith JR, Chaignaud B, Schamberger RC, Huang SA (2011). Thyroid surgery at Children’s Hospital Boston: a 35-year single-institution experience. J Pediatr Surg.

[6] Hanba C, Svider PF, Siegel B, Sheyn A, Shkoukani M, Lin HS, Raza SN (2017). Pediatric thyroidectomy: hospital course and perioperative complications. Otolaryngol Head Neck Surg.

[7] Sosa JA, Tugle CT, Wang TC (2008). Clinical and economic outcomes of thyroid and parathyroid surgery in children. J Clin Endocriniol Metab.

[8] Klein Hesselink MS, Nies M, Bocca G, Brouwers AH, Burgerhof JG, van Dam EW, Havekes B, van den Heuvel-Eibrink MM, Corssmit EP, Kremer LC, Netea-Maier RT, van der Pal HJ, Peeters RP, Schmid KW, Smit JW, Williams GR, Plukker JT, Ronckers CM, van Santen HM, Tissing WJ, Links TP (2016). Pediatric differentiated thyroid carcinoma in The Netherlands: a nationwide follow-up study. J Clin Endocrinol Metab.

[9] Scandinavian quality register for thyroid parathyroid and adrenal surgery. Annual report 2016. www.sqrtpa.se

[10] Wettermark B, Hammar N, Fored CM, Leimanis A, Otterblad Olausson P, Bergman U, Persson I, Sundström A, Westerholm B, Rosén M (2007). The new Swedish prescriped drug register—opportunities for pharmacoepidemiological research and experience from the first six months. Pharmacoepidemiol Drug Saf.

[11] National Board of Health Sweden. http://www.socialstyrelsen.se/register/halsodataregister/patientregistret/inenglish. Accessed October 2017

[12] Bron LP, O’Brien CJ (2004). Total thyroidectomy for clinically benign disease of the thyroid gland. Br J Surg.

[13] Efremidou E, Papageorgiou MS, Liratzopoulos N, Manolas KJ (2009). The efficacy and safety of total thyroidectomy in the management of benign thyroid disease: a review of 932 cases. Can J Surg.

[14] Bargren AE, Meyer-Rochow GY, Delbridge LW, Sidhu SB, Chen H (2009). Outcomes of surgically managed pediatric thyroid cancer. J Surg Res.

[15] Hauch A, Al-Qurayshi Z, Randolph G (2014). Total thyroidectomy is associated with increased risk of complications for low- and high-volume surgeons. Ann Surg Oncol.

[16] Sosa JA, Bowman HM, Tielsch JM (1998). The importance of surgeon experience for clinical and economic outcomes from thyroidectomy. Ann Surg.

[17] Kandil E, Noureldine SI, Abbas A (2013). The impact of surgical volume on patient outcomes following thyroid surgery. Surgery.

[18] Adam MA, Thomas S, Youngwirth L, Hyslop T, Reed SD, Scheri RP, Roman SA, Sosa JA (2017). Is there a minimum number of thyroidectomies a surgeon should perform to optimize patient outcomes?. Ann Surg.

[19] Edafe O, Antakia R, Laskar N, Uttley L, Balasubramanian SP (2014). Systematic review and meta-analysis of predictors of post-thyroidectomy hypocalcaemia. Br J Surg.

[20] Antakia R, Edafe O, Uttley L, Balasubramanian SP (2015). Effectiveness of preventative and other surgical measures on hypocalcemia following bilateral thyroid surgery: a systematic review and meta-analysis. Thyroid.

[21] Falco J, Dip F, Quadri P, de la Fuente M, Prunello M, Rosenthal RJ (2017). Increased identification of parathyroid glands using near infrared light during thyroid and parathyroid surgery. Surg Endosc.

[22] Lorente-Poch L, Sancho J, Muñoz JL, Gallego-Otaegui L, Martínez-Ruiz C, Sitges-Serra A (2017). Failure of fragmented parathyroid gland autotransplantation to prevent permanent hypoparathyroidism after total thyroidectomy. Langenbecks Arch Surg.

